# Rare early hematogenous disseminated tuberculosis inducing hemophagocytic syndrome in conflict treatment

**DOI:** 10.1186/s12879-025-11278-7

**Published:** 2025-07-23

**Authors:** Xiao-Hui Jin, Xin-Wei Shang, Hui-Qiang Zhang

**Affiliations:** The First Affiliated Hospital of Xinxiang Medical University, Xinxiang, China

**Keywords:** Hematogenous disseminated pulmonary tuberculosis, Hemophagocytic syndrome, Cytokine storm, Immunosuppressive therapy

## Abstract

This article presents a case of an acute onset in a middle-aged male who exhibited persistent high fever (temperature > 40 °C), delirium, and respiratory distress. Initial chest CT only suggested "bilateral pneumonia," and empirical anti-infection treatment proved ineffective. The patient subsequently developed pancytopenia, splenomegaly, and markedly elevated ferritin levels (848.90 μg/L). Bone marrow aspiration demonstrated hemophagocytic activity and granulomatous lesions. A positive TB-PCR, confirmed the diagnosis of early hematogenous disseminated tuberculosis complicated by the hemophagocytic syndrome (HLH). The patient's condition gradually improved Following, individualized anti-tuberculosis therapy and immunosuppressive treatment. The uniqueness of this case lies in two main aspects: (1) early imaging did not show typical miliary nodules, which could have led to misdiagnosis as common pneumonia; (2) the dissemination of tuberculosis and onset of HLH occurred almost simultaneously, creating a therapeutic dilemma. Although tuberculosis complicated by HLH is rare, it poses significant danger. The cornerstone of treatment is effective control of the tuberculosis infection while simultaneously suppressing the excessive immune response. This case highlights the importance of considering tuberculosis complicated by HLH in patients with recurrent fever and pulmonary infiltrates. Early diagnosis and prompt treatment are crucial for improving prognosis. The article also discusses the underlying pathogenesis, offering valuable insights for clinical practice.

**Supplementary Information:**

The online version contains supplementary material available at 10.1186/s12879-025-11278-7.

## Introduction

Hemophagocytic syndrome (HPS), also known as hemophagocytic lymphohistiocytosis (HLH), is a group of life-threatening clinical syndromes characterized by a cytokine storm. It typically present with symptoms such as fever, splenomegaly, cytopenia, elevated ferritin levels, and liver dysfunction [1]. This is a rare disease, with an estimated incidence of approximately 1 in 800,000 adults per year and 1 to 10 per million in children. The disease progresses rapidly and is associated with a high mortality rate [2, 3]. Common secondary causes include infections, malignancies, autoimmune diseases, and iatrogenic factors. However, cases associated with or secondary to tuberculosis (TB) are relatively rare [4]. Here, we report a case of hematogenous disseminated tuberculosis (HDT) complicated by HPS treated at our hospital, aiming to raise clinicians’ awareness of this potentially fatal condition.

## Case information

The patient is a 52-year-old male worker who was admitted to the hospital on April 27, 2024, due to a three-day history of fever. Three days prior to admission, he developed a fever with a maximum temperature of 40 °C, accompanied by delirium, chills, rigors, night sweats, chest tightness, shortness of breath, palpitations, and bilateral lower limb edema, along with urinary urgency. He initially sought treatment at a local hospital, where chest CT revealed bilateral pneumonia. He received cefoperazone for three days, but the response was poor, and he was urgently transferred to our hospital. On physical examination, his vital signs were: T 38.8 °C, P 101 beats/min, R 32 breaths/min, BP 142/96 mmHg, and SpO₂ 89%. He appeared acutely ill, had a dull facial expression, and was in a state of delirium. Bilateral coarse breath sounds were heard, accompanied by fine crackles at the lung bases and scattered rhonchi in the upper lobes. Mild pitting edema was present in both lower limbs. Pathological reflexes were absent. The patient had a history of untreated hypertension and a long-term history of alcohol consumption. He denied any known close contact with tuberculosis patients.

**Fig. 1:**
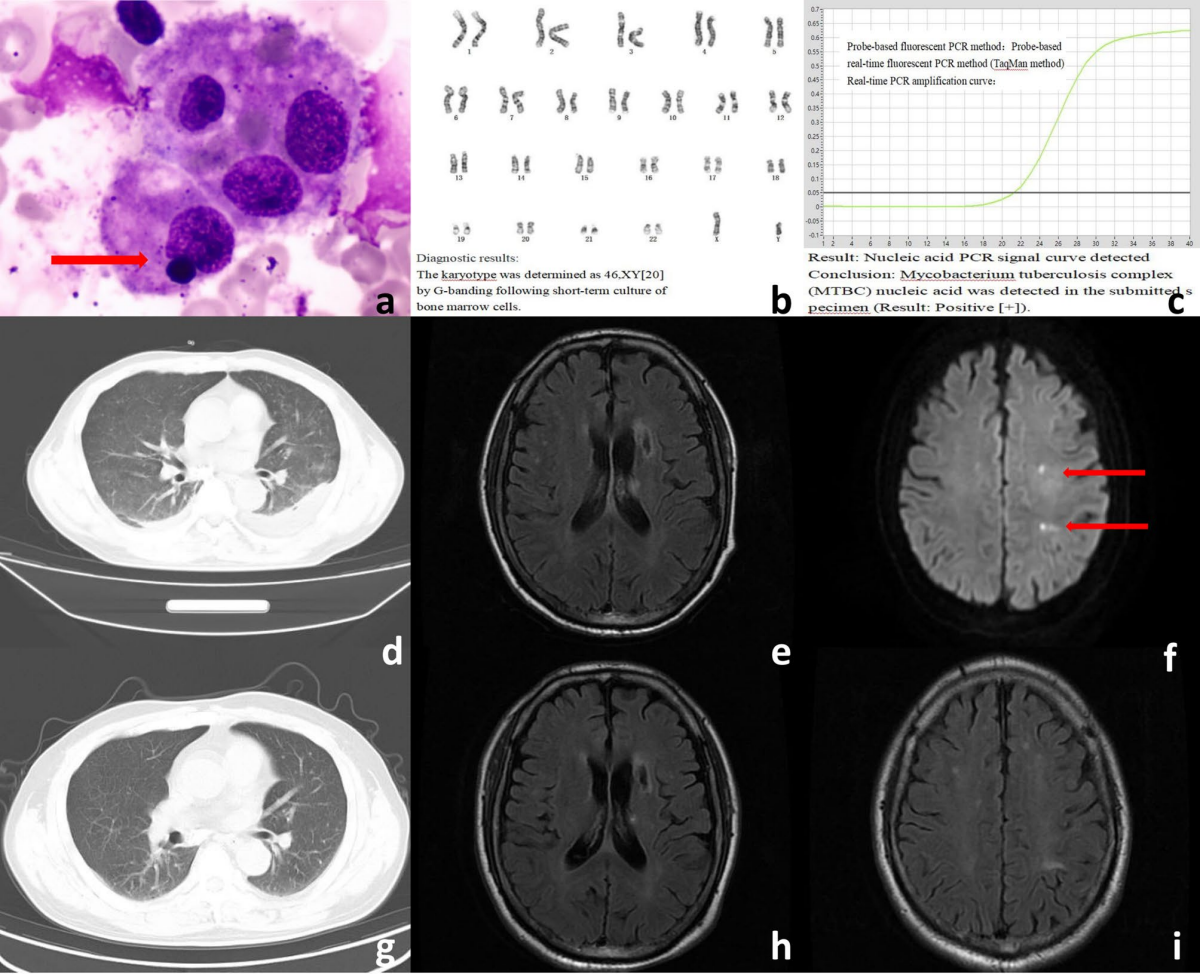
**a** Bone marrow cytology report demonstrating phagocytic phenomena. **b** Bone marrow cytology report demonstrating phagocytic phenomena. **c** Bone marrow pathology TB-DNA PCR test result: positive. **d** Chest CT (2024–05-07) showing diffuse, multiple punctate shadows in both lungs, along with bilateral pleural effusion. **e**–**f** Cranial MRI (2024–05-15) showing multiple abnormal signals and partial enhancement in the bilateral periventricular region, left frontal lobe, basal ganglia, and left temporal lobe. **g** Chest CT (2024–11-30) showing a reduction in punctate and small nodular lesions in both lungs compared to earlier scans. **h**-**i** Cranial MRI (2024–11-30) demonstrating marked reduction and shrinkage of abnormal enhancement areas compared to prior imaging

Admission tests showed the following results: arterial blood gas analysis revealed a pH of 7.44, FiO₂ 29% (via 2 L/min nasal cannula), bicarbonate 20.4 mmol/L, PaO₂ 51 mmHg, PaCO₂ 27.1 mmHg, and SpO₂ 87.7%. Coagulation tests showed a D-dimer lever of 8.11 µg/mL, and C-reactive protein (CRP) was elevated at 57.80 mg/L. Blood biochemistry results were as follows: sodium 128 mmol/L, calcium 1.77 mmol/L, albumin 27.6 g/L, alanine aminotransferase (ALT) 78 U/L, aspartate aminotransferase (AST) 139 U/L, and gamma-glutamyl transferase (GGT) 166 U/L. Fungal D-glucan was elevated at 251.95 pg/mL. Lymphocyte subset analysis showed CD3⁺CD4⁺ lymphocytes at 177.65 cells/µL. Routine tests of blood, urine, and stool, along with respiratory pathogen screening, antinuclear antibody (ANA) spectrum, thyroid function, and B-type natriuretic peptide (BNP), revealed no significant abnormalities. Diagnostic workup showed negative results for acid-fast bacilli smear, HIV antibody and nucleic acid tests, and quantitative PCR for Epstein-Barr virus (EBV) DNA and cytomegalovirus (CMV) DNA. The electrocardiogram (ECG) indicated sinus tachycardia. Abdominal ultrasound revealed splenomegaly. Chest CT demonstrated bilateral pleural effusions, pulmonary atelectasis, diffuse and multifocal nodular opacities in both lungs, and splenomegaly (Fig. 1d).

Diagnostic Dilemma: The patient, who presented with acute infection upon admission and had demonstrated a suboptimal response to prior antimicrobial therapy at the local hospital, was initiated on a 10-day course of broad-spectrum antimicrobial therapy. The regimen included intravenous cefoperazone-sulbactam (3 g every 8 h) combined with moxifloxacin (400 mg once daily) for bacterial coverage. Notably, caspofungin (50 mg once daily) was added for antifungal coverage, given the elevated Fungal D-glucan level (251.95 pg/mL) and the potential of fungal infection. Supportive therapies included nebulized bronchodilators and mucolytic agents for respiratory symptoms, supplemental oxygen, proton pump inhibitors (pantoprazole 40 mg twice daily) for gastric protection, and both enteral and parenteral nutrition to meet metabolic demands. Despite these interventions, the patient continued to have a persistent fever (peak temperature 38.9 °C), with negative results from blood cultures (aerobic and anaerobic) and respiratory viral PCR testing. The infection source remained unidentified. Due to the suboptimal clinical response, the antimicrobial regimen was escalated to meropenem (1 g every 8 h, IV infusion) combined with tigecycline (100 mg loading dose followed by 50 mg every 12 h), while caspofungin (50 mg daily) was maintained. This optimized antimicrobial therapy was administered for 7 days. As the patient continued to experience high fever along with rapidly progressing cytopenia (platelet count dropping to 28 × 10⁹/L), HLH was considered. Emergency bone marrow aspiration and infusion therapy with platelets and hematopoietic growth factors were initiated. Peripheral blood smear showed: 1) A significant decrease in white blood cells,; 2) A reduced granulocyte ratio; 3) Immature red blood cells of varying sizes (50 white blood cells counted; no nucleated red blood cells observed); 4) An increased lymphocyte ratio; 5) Mild scattered platelets, with some large platelets visible. Bone marrow cytology indicated active marrow proliferation with normal development across all hematopoietic lineages, but phagocytic phenomena were observed. Reticulocytes accounted for 1.5% (Fig. 1a). Bone marrow cytogenetic analysis showed that G-banding performed after a short-term culture revealed a karyotype of 46, XY[20] (Fig. 1b). Following a multidisciplinary team (MDT) consultation, the diagnosis of HLH was confirmed, though HDT could not be excluded. Further investigation with enhanced cranial MRI showed multiple abnormal enhancements in the brain (Fig. 1e-f). Cerebrospinal fluid (CSF) analysis, including routine and biochemical testing, showed no significant abnormalities. Xpert MTB/RIF was negative. CSF cytology revealed 48% lymphocytes, 50% monocytes, and 2% activated lymphocytes, with all other parameters at 0. A bone marrow biopsy revealed granulomatous lesions, possibly of tuberculosis origin. Acid-fast staining was negative, but PCR for TB-DNA was positive (Fig. 1c). The final diagnosis was hematogenous disseminated tuberculosis, tuberculous meningitis, and secondary hemophagocytic syndrome.

Challenges in early tuberculosis diagnosis were evident in this case.Upon admission, the patient’s chest CT only showed patchy shadows in both lungs and pleural effusion, lacking the characteristic “millet-like nodules” typically seen in HDT, which led to an initial misdiagnosis of pulmonary infection. It was only after disease progression (on day 10) and repeat imaging that diffuse miliary nodules were observed. Early sputum smear and Xpert MTB/RIF tests were negative, and the final diagnosis relied on TB-PCR from a bone marrow biopsy, highlighting the critical importance of deep tissue sampling in suspected disseminated TB. Moreover, the patient’s initial clinical picture was dominated by symptoms of HLH—such as persistent fever and cytopenia—without typical pulmonary symptoms like cough, which contributed to diagnostic confusion with hematologic malignancies or severe sepsis.

Treatment posed additional challenges, as standard anti-tuberculosis drugs are known for their hepatotoxicity and bone marrow suppression, while this patient already exhibited elevated liver enzymes and pancytopenia at baseline. Therefore, a carefully individualized regimen was initiated, invluding isoniazid (300 mg/day) for cell wall synthesis inhibition, levofloxacin (750 mg/day) for DNA replication blockade, amikacin (15 mg/kg/day) and contezolid (800 mg twice daily) for protein synthesis inhibition, and ethambutol (15 mg/kg/day) for arabinogalactan synthesis disruption. To control HLH, the patient was treated according to the HLH-94 protocol, combining etoposide (150 mg/m^2^ weekly) and dexamethasone (10 mg/m^2^/day with tapering), supplemented with the JAK1/2 inhibitor ruxolitinib (10 mg twice daily) to suppress cytokine overproduction via JAK-STAT pathway inhibition. Supportive care measures included hepatoprotective therapy, hematopoietic growth factor support, and prophylaxis antimicrobial treatment. This comprehensive approach simultaneously targeted both Mycobacterium tuberculosis infection and the immune dysregulation of HLH, while minimizing drug toxicity through close monitoring. Fellowing trearment initiation, the patient's clinical condition gradually stabilized, and his body temperature returned to normal.

**Fig. 2:**
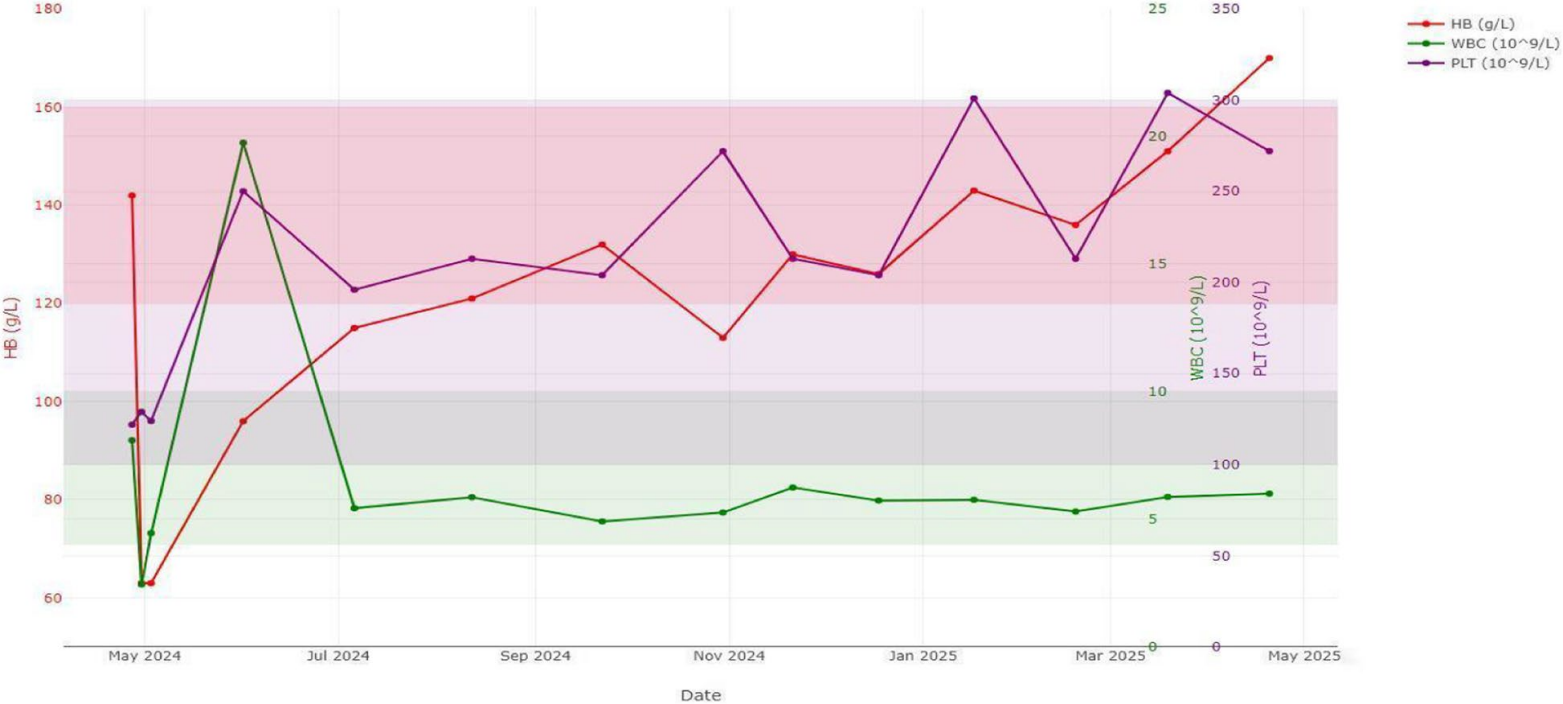
Trends in WBC, PLT, and Hb

**Fig. 3:**
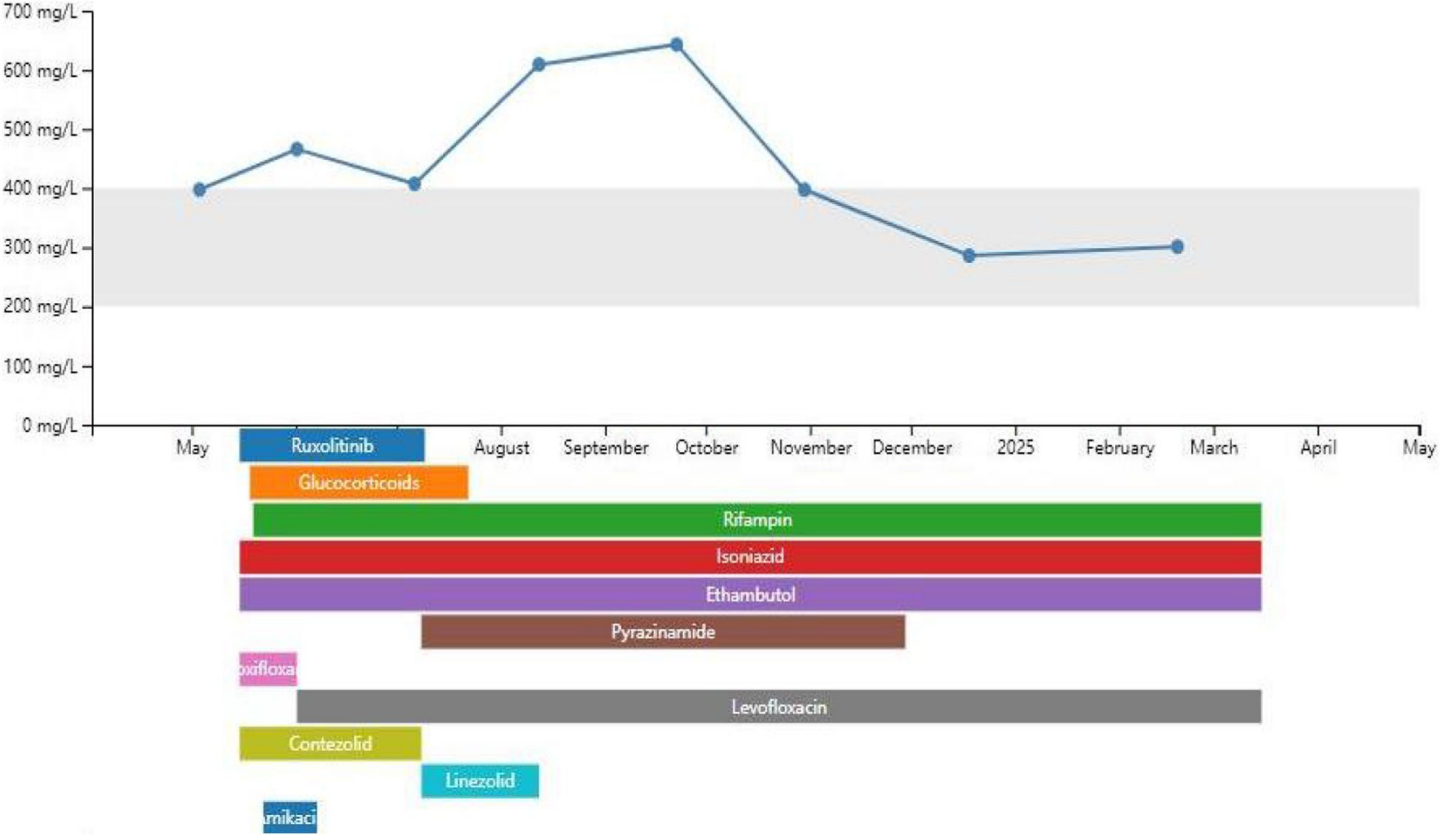
Trends in cerebrospinal fluid total protein and treatment regimen

**Fig. 4:**
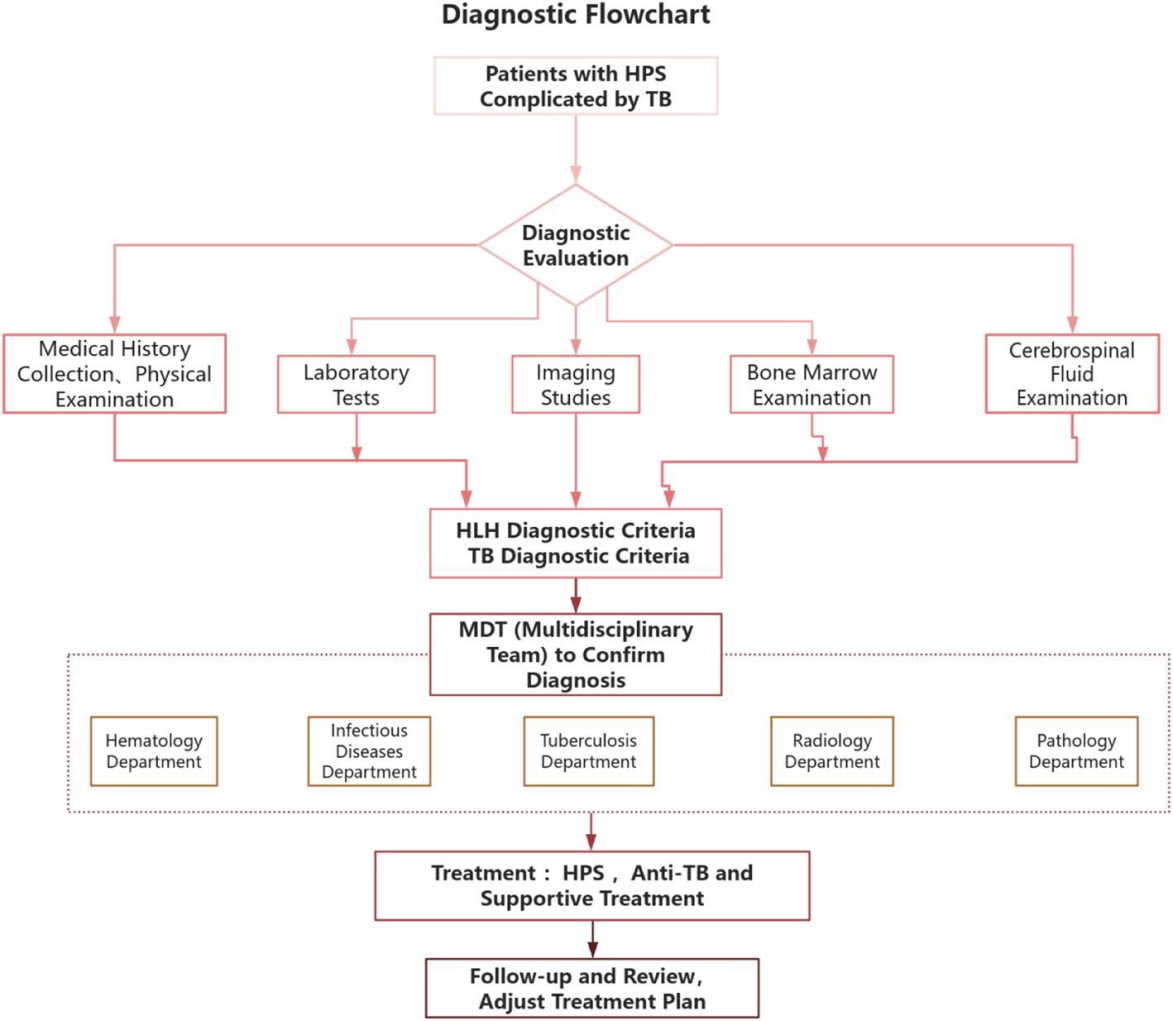
Diagnostic flowchart of hematogenously disseminated tuberculosis complicated by hemophagocytic lymphohistiocytosis (HLH). Note: Key decision points include: if fever persists for more than 7 days and is accompanied by pancytopenia, HLH should be promptly ruled out through tests such as ferritin level assessment and bone marrow aspiration; if imaging results are inconclusive, broaden the scope of pathogen detection (e.g., bone marrow TB-PCR, lymph node biopsy); in the early phase of treatment, priority should be given to controlling the HLH-related cytokine storm, followed by the gradual intensification of anti-tuberculosis therapy

**Fig. 5:**
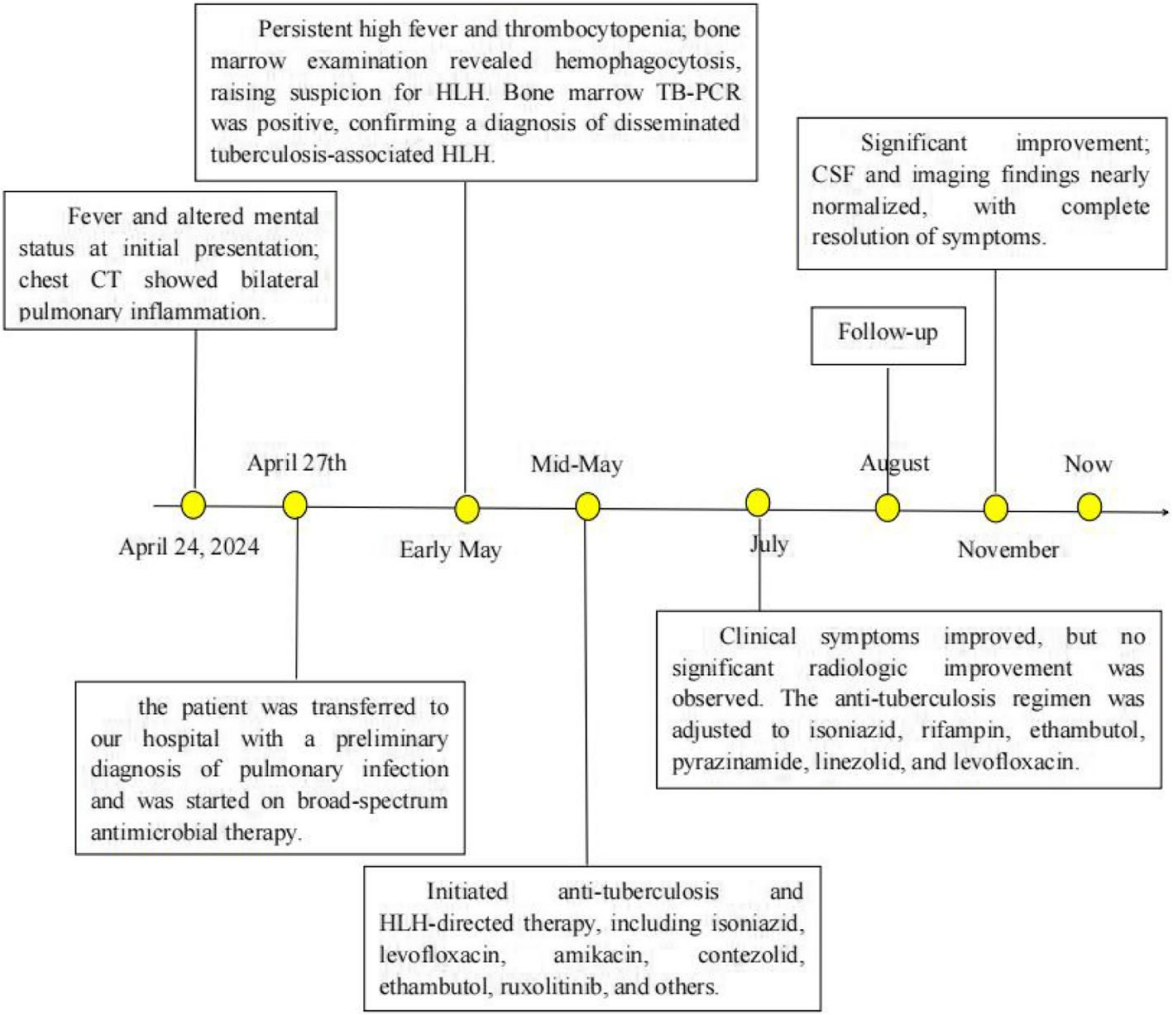
Clinical timeline of diagnosis, treatment, and follow-up

Follow-up: In July 2024, the patient returned for follow-up. Hematologic and liver function tests had improved, and symptoms of HLH had subsided. Ruxolitinib was gradually discontinued. Despite the clinical improvement, chest CT revealed increased millet-like and patchy high-density opacities in both lungs, and cranial MRI showed enlarged areas of abnormal enhancement. CSF protein was mildly elevated at 609.30 mg/L. Nevertheless, given the overall stabilization of hematologic (Fig. 2) and hepatic parameters, the anti-tuberculosis regimen was de-escalated to a six-drug combination: isoniazid (300 mg daily), rifampin (600 mg daily), ethambutol (15 mg/kg daily), pyrazinamide (25 mg/kg daily), linezolid (600 mg every 12 h), and levofloxacin (750 mg daily) (Fig. 3). At the follow-up in August 2024, partial absorption of pulmonary lesions and reduction in intracranial abnormalities were observed, indicating an initial therapeutic response. By November 2024, imaging showed significant improvement: chest CT revealed marked resolution of pulmonary lesions (Fig. 1g), and cranial MRI demonstrated substantial shrinkage of intracranial abnormalities (Fig. 1 h-i). CSF parameters had normalized, and the patient’s symptoms had completely resolved. As of now, the patient has completed 11 months of ongoing anti-tuberculosis therapy with the current regimen. Treatment of hematogenous disseminated tuberculosis typically requires a minimum duration of 12 months. Outpatient follow-up will continue to monitor for potential recurrence, and treatment discontinuation will depend on radiographic and microbiological confirmation of success (Figs. 4 and 5).

## Discussion

### The Relationship Between TB and HLH

TB is a chronic infectious disease caused by Mycobacterium tuberculosis (MTB). While its incidence has significantly declined in China, the country still ranks among the top three globally in terms of total case numbers, making it one of the world’s highest TB-burden regions [5]. In recent years, significant advances in pulmonary imaging have greatly improved diagnostic accuracy, especially for early-stage and disseminated TB. Non-invasive techniques such as hyperpolarized gas magnetic resonance imaging (HP-MRI) are emerging as promising alternatives to conventional chest X-rays and CT scans, which are limited by radiation exposure and insufficient sensitivity to subtle lesions. Novel contrast agents, including hyperpolarized diethyl ether and butane, have abled high-resolution, functional imaging of pulmonary ventilation, offering new avenues for evaluating treatment response in TB patients [6–8].

Delayed or inadequate treatment of TB can lead to severe complications involving multiple organ systems. Among these, HLH is a particularly severe and frequently underrecognized outcome. HLH secondary to TB, especially in cases of HDT, has been increasingly reported in clinical practice. HDT occurs when MTB spreads widely through the bloodstream, typically affecting individuals with compromised immunity, liver cirrhosis, or metabolic comorbidities [9]. Clinically, HDT may present with respiratory symptoms or systemic features such as hepatosplenomegaly, cytopenias, headache, and vomiting. Many of these manifestations overlap with those of HLH, thereby complicating early recognition diagnosis. Although HDT accounts for only 1–2% of all TB cases, it carries a high mortality rate of up to 90%, particularly when complicated by HLH [10].

At the molecular level, TB-related immune dysregulation involves both oxidative and inflammatory pathways, particularly those mediated by nitric oxide (NO) and tetrahydrobiopterin (BH4). Although BH4 deficiency in macrophages reduces NO production, it may paradoxically enhance MTB clearance via NO-independent mechanisms, including altered lysosomal activity and shifts in inflammatory signaling. Conversely, oxidative stress—primarily driven by reactive oxygen species such as peroxynitrite—can degrade GTP cyclohydrolase I (GCH1), leading to BH4 depletion and impaired macrophage function. These dual roles of BH4 highlight a delicate immunological balance: while reduced BH4 may aid bacterial control, oxidative loss of BH4 can intensify tissue damage and immune overactivation, contributing to HLH pathogenesis in severe TB cases [11, 12].

HLH secondary to HDT typically progresses rapidly and frequently presents initially with unexplained fever [13], along with hematologic abnormalities such as bicytopenia or pancytopenia [14], skin rash, and other nonspecific systemic symptoms [15]. The underlying pathogenesis involves abnormal activation of the monocyte-macrophage system, leading to excessive production of pro- inflammatory cytokines (such as tumor necrosis factor-α), suppression of bone marrow hematopoiesis, and hemophagocytosis [16, 17].This dysregulated immune response is closely linked to oxidative stress mechanisms associated with BH4 imbalance, which further anplifies disease severity. As such, TB-associated HLH represents a critical intersection between infectious and immune dysfunction, requiring heightened clinical vigilance and a multidisciplinary approach to management.

### The pathological mechanism of HLH

HLH is a rare is a rare but life-threatening syndrome characterized by excessive activation of cytotoxic T lymphocytes, natural killer (NK) cells, and macrophages. This immune hyperactivation leads to a massive release of pro-inflammatory cytokines—a so-called"cytokine storm"—that triggers a cascade of clinical manifestations, culminating in multiorgan damage and, in many cases, death [18]. HLH arises from abnormal activation of the immune system, which may be caused by either primary (genetic) defects or secondary (acquired) triggers, resulting in an uncontrolled inflammatory response. The estimated annual incidence is approximately 1 in 800,000 individuals [19]. Despite its rarity, HLH is associated with a high mortality rate, ranging from 50 to 70% [20]. Without treatment, the median survival time is less than two months [21–23]. Primary HLH is an autosomal recessive genetic disorder caused by mutations affecting genes that regulate immune homeostasis, leading to impaired cytotoxic function and persistent inflammation. It typically manifests in infancy or early childhood, with an incidence of about 1 in 800,000 per year. In contrast, secondary HLH is triggered by external stimuli such as infections, malignancies, autoimmune conditions, or iatrogenic factors [24]. Among infection-associated secondary HLH cases, Epstein-Barr virus (EBV) is the most commonly implicated pathogen, while tuberculosis-associated HLH is less frequently reported but increasingly recognized in clinical settings [25]. HLH is typically fulminant and rapidly progressive. The primary causes of mortality include disseminated intravascular coagulation (DIC), hemorrhagic complications, severe infections, and multiple organ failure [26].

### TB-Associated HLH: diagnostic challenges

The pathophysiological mechanism by which TB induces secondary HLH remains incompletely elucidated. However, emerging evidence suggests that MTB infection can initiate a hyperinflammatory state through multiple immune pathways. In clinical settings, the presence of persistent high-grade fever, cytopenia, splenomegaly, and elevated inflammatory markers often raises early suspicion for HLH.While a cytokine storm and macrophage overactivation are hallmark features of HLH [27–29], TB may further intensify these immune responses through dysregulation of immune homeostasis—particularly involving Th1/Th2 imbalance and impaired bacterial clearance [30]. Additionally, the “cross-reactive antigen” hypothesis has been proposed to explain hematologic abnormalities observed in TB-associated HLH, suggesting that molecular mimicry between MTB antigens and host hematopoietic components may contribute to immune-mediated cytopenias [3, 31].

This patient presented with overlapping features of HDT and HLH, with a definitive diagnosis ultimately confirmed via bone marrow biopsy. HDT is frequently associated with tuberculous meningitis (TBM) [32], and in the context of immune exhaustion, CSF testing in TBM patients may yield false-negative results. In such cases, MRI offers superior diagnostic sensitivity. The triad of miliary pulmonary tuberculosis, HLH, and TBM represents an extremely rare but highly fatal clinical condition. TBM can further exacerbate immune dysregulation, particularly within the central nervous system. Shared clinical manifestations—such as persistent fever, cytopenia, and increased intracranial pressure—often obscure diagnostic boundaries between these conditions, leading to delayed treatment.

Effective management of TB-associated HLH requires timely recognition and a comprehensive treatment approach, including anti-tuberculosis therapy, HLH-targeted interventions (such as corticosteroids and etoposide), and supportive measures to manage elevated intracranial pressure [33]. However, immunosuppressive therapy may paradoxically facilitate further dissemination of *Mycobacterium tuberculosis*. This case underscores three important clinical considerations: (1) Patients with miliary TB should routinely undergo contrast-enhanced cranial MRI to screen for concurrent TBM; (2) Persistent unexplained fever and cytopenia should raise suspicion for underlying HLH; (3) Multidisciplinary coordination is crucial to balancing antimicrobial therapy, immunosuppression, and supportive care in order to optimize patient outcomes.

### HLH Diagnosis

According to the 2022 Chinese Expert Consensus on HLH, a diagnostic can be established based on either of two sets of criteria (Table 1) [22]. In this case, the patient presented with a persistent fever lasting nearly one month prior to a definitive diagnosis of tuberculosis. The diagnostic delay was primarily due to atypical clinical manifestations and nonspecific initial chest imaging, which led to a transient resolution of fever symptoms. This, in turn, masked the underlying condition and interfered with timely clinical judgment. As the disease progressed, the patient developed splenomegaly and pancytopenia, with a particularly significant decline in platelet count, raising clinical suspicion of a hematologic disorder. Subsequent bone marrow aspiration and biopsy confirmed a tuberculosis infection and revealed hemophagocytic activity in the marrow, along with elevated serum ferritin and hypofibrinogenemia—meeting six of the eight diagnostic criteria outlined in Table 1. Potential causes of secondary HLH, including malignancies (such as leukemia, lymphoma, and other solid tumors), rheumatic autoimmune diseases, Epstein-Barr virus (EBV), and other known infections, were excluded. After ruling out primary HLH and other causes seconfary causes, a final diagnosis of tuberculosis-associated HLH was established.

**Table 1:** 2022 Chinese expert consensus on hemophagocytic lymphohistiocytosis (HLH) diagnostic criteria

The diagnosis of hemophagocytic lymphohistiocytosis (HLH) can be made if any of the following two criteria are met		Compare with this case
(1) The diagnosis can be made by identifying HLH-related genetic abnormalities, combined with clinical findings	Such genetic mutations include PRF1, UNC13D, STX11, STXBP2, Rab27a, LYST, SH2D1A, BIRC4, ITK, AP3B1, MAGT1, CD27, and others	Not tested
(2) Meets 5 out of the following 8 criteria		Meets 6 out of the 8 criteria
①Fever	A temperature > 38.5 °C, lasting for more than 7 days	Abnormal (maximum temperature of 38.9 °C, lasting for 16 days)
②splenomegaly	The spleen can be palpated more than 3 cm below the left costal margin	Abnormal (splenomegaly visible on ultrasound)
③Pancytopenia (involving two or three cell lines in peripheral blood)	Hemoglobin < 90 g/L, platelet count < 100 × 10^9/L, neutrophils < 1.0 × 10^9/L, caused by impaired extramedullary hematopoiesis	Abnormal (hemoglobin 61 g/L, platelet count 64 × 10^9/L, neutrophils 0.93 × 10^9/L)
④Hypertriglyceridemia and/or hypofibrinogenemia	Triglycerides > 3 mmol/L or more than 3 standard deviations above the age-matched norm, fibrinogen < 1.5 g/L or more than 3 standard deviations below the age-matched norm	Abnormal (fibrinogen 1.2 g/L)
⑤Hemophagocytic cells	In the bone marrow, spleen, liver, or lymph nodes	Abnormal (hemophagocytic cells found in bone marrow images)
⑥Elevated serum ferritin	Ferritin > 500 μg/L	Abnormal (ferritin 848.90 μg/L)
⑦NK cell activity	Reduced or absent	Not examined
⑧Elevated sCD25	≥ 2400 U/ml	Not examined

Chinese Society of Hematology, Chinese Pediatric Society Hematology Group, Hemophagocytic Lymphohistiocytosis Chinese Expert Alliance. Chinese Guidelines for Diagnosis and Treatment of Hemophagocytic Lymphohistiocytosis (2022 Edition) [M]. 2022

### Treatment strategy

The cornerstone of treating tuberculosis-associated HLH lies in controlling MTB infection and suppressing the excessive immune response. Standard management typically includes anti-tuberculosis therapy, immunosuppressive treatment, and supportive care. According to the *Chinese Guidelines for the Diagnosis and Treatment of Hemophagocytic Lymphohistiocytosis* (2022 Edition), the *Tuberculosis Diagnosis and Treatment Guidelines* (2019 Edition), and the *American Society of Hematology (ASH) HLH Treatment Guidelines* (2019 Edition) [17, 22, 34], anti-tuberculosis therapy is essential, with commonly used agents including isoniazid, rifampin, pyrazinamide, and ethambutol. For immunosuppressive therapy, corticosteroids (such as dexamethasone) and etoposide are considered first-line treatments. Corticosteroids exert anti-inflammatory effects by inhibiting the NF-κB pathway and reducing the release of pro-inflammatory cytokines [35], whereas etoposide promotes apoptosis of overactivated T cells and macrophages, thereby mitigating the cytokine storm [36]. In cases of refractory HLH, novel immunomodulatory agents have shown promising efficacy. These include the JAK inhibitor ruxolitinib and IL-1 receptor antagonists such as anakinra. Ruxolitinib suppresses the inflammatory cascade by inhibiting the JAK-STAT pathway and reducing the production of IFN-γ and IL-6 [37], while IL-1 receptor antagonists alleviate inflammation and tissue damage by blocking IL-1 signaling [17]. As understanding of HLH immunopathogenesis has advanced, a growing number of targeted therapies have emerged. For example, the anti-IFN-γ monoclonal antibody emapalumab has demonstrated efficacy in both primary and refractory HLH [38]. Additionally, inflammasome inhibitors such as MCC950 have shown therapeutic potential in preclinical HLH models [39].

### Treatment contradictions and solutions

Drug Toxicity Overlap: The hepatotoxicity of first-line anti-tuberculosis drugs (such as rifampin and pyrazinamide) poses a significant risk to HLH patients with concurrent liver dysfunction. An individualized treatment plan should be adopted in the early stages of the disease. Initially, hepatotoxic drugs should be avoided; instead, agents with minimal bone marrow suppression, such as levofloxacin and amikacin, can be combined with low-dose corticosteroids to control HLH. Once the condition stabilizes, rifampin and isoniazid can be gradually introduced, with dosages adjusted dynamically via therapeutic drug monitoring (TDM), while closely monitoring liver enzymes and complete blood counts. Conflict of Immunotherapy: The treatment of HLH requires rapid immunosuppression (such as etoposide and high-dose corticosteroids), which may compromise the anti-tuberculosis immune response. In this case, ruxolitinib—a novel JAK inhibitor—effectively reduced IFN-γ levels without inducing tuberculosis exacerbation, offering a new strategy to balance immunosuppression with infection control. Treatment Regimen in This Case: The patient initially used anti-tuberculosis drugs with minimal impact on the blood system and liver function: isoniazid, ethambutol, amikacin, levofloxacin, and contezolid, forming an individualized treatment regimen. This was combined with corticosteroids and ruxolitinib for immunotherapy. Once HLH improved, rifampin, isoniazid, ethambutol, and pyrazinamide were introduced to enhance the anti-tuberculosis effect, and linezolid replaced etizolam to reduce the patient's financial burden [40].

## Conclusion

Although tuberculosis-associated hemophagocytic lymphohistiocytosis (HLH) is relatively rare, it is a life-threatening condition that warrants a high level of clinical vigilance. This case demonstrates that hematogenous disseminated tuberculosis (HDT) may initially present with only nonspecific systemic inflammatory signs, with HLH serving as an important clinical warning signal. Timely diagnostic procedures, such as bone marrow aspiration, are critical for confirming the diagnosis, particularly when conventional tests fail to provide definitive results. Effective treatment relies on a combined strategy of anti-tuberculosis therapy and immunosuppressive management, with close monitoring required to balance infection control and immune regulation. A"host-directed"anti-tuberculosis approach incorporating novel JAK inhibitors may improve clinical outcomes.This case highlights several key clinical insights: (1) HLH may be the initial manifestation of disseminated tuberculosis, especially when other causes are excluded.(2) Multidisciplinary collaboration is essential for achieving favorable outcomes in such complex immuno-infectious syndromes.(3) The use of immunosuppressive agents should be supported by effective anti-tuberculosis therapy. These insights provide valuable guidance for the clinical management of this critical condition.

## Supplementary Information

Supplementary Material 1.

## Data Availability

The datasets used during the current study are available from the corresponding or last author on reasonable request.

## Abbreviations

HLH: Hemophagocytic lymphohistiocytosis
TB: Tuberculosis
HDT: Hematogenous disseminated tuberculosis
ALT: Alanine aminotransferase
AST: Aspartate aminotransferase
GGT: Gamma-glutamyl transferase
MDT: Multidisciplinary team
WBC: White blood cell
PLT: Platelet
Hb: Hemoglobin
