# Supplementary material for: Rare early hematogenous disseminated tuberculosis inducing hemophagocytic syndrome in conflict treatment
Source: BMC Infect Dis. 2025 Jul 23;25:935. doi: 10.1186/s12879-025-11278-7 (PMC12285026; doi:10.1186/s12879-025-11278-7)
Supplement: Supplementary file 1 — Supplementary Material 1. [file 12879_2025_11278_MOESM1_ESM.pdf]

Supplementary Material

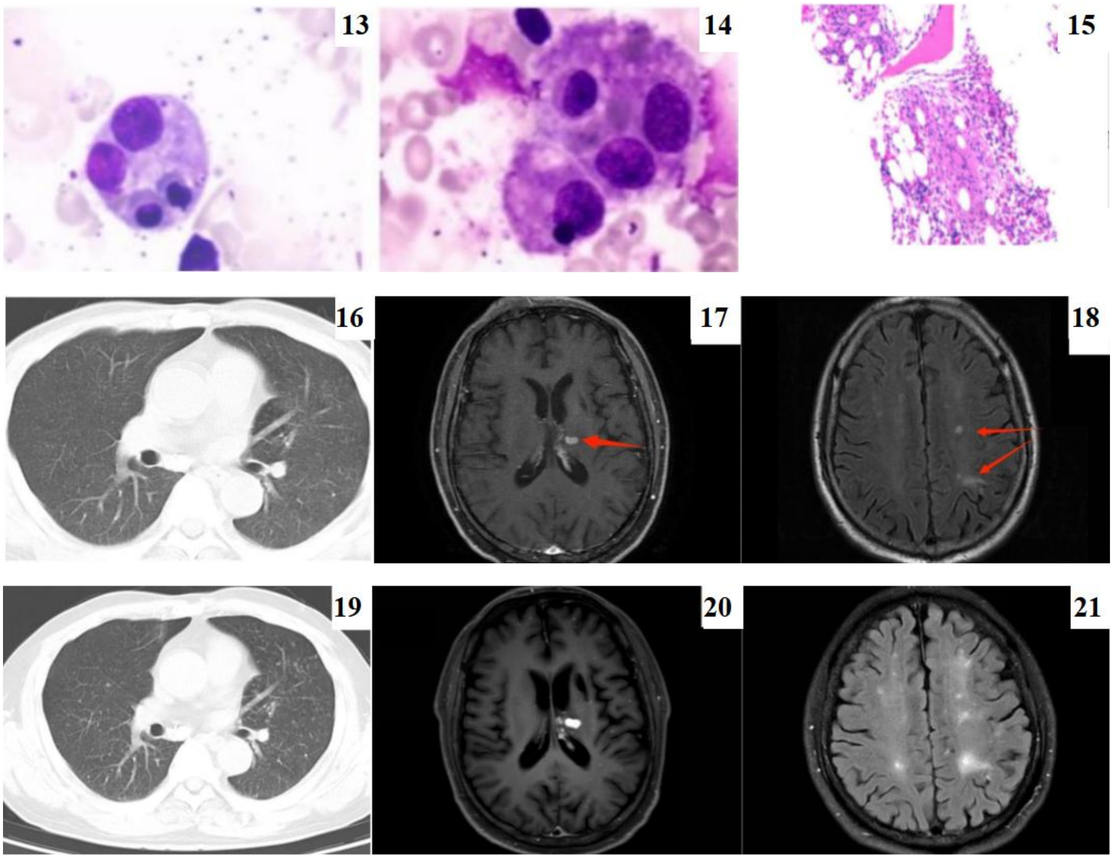

Figure 13-14: Bone marrow cytology report demonstrating phagocytic phenomena.

Figure 15: Bone marrow pathology report showing granulomas.

Figure 16: Chest CT (2024-06-06) showing an increase in the number of diffuse, multiple punctate shadows in both lungs compared to previous images.

Figures 17-18: Cranial MRI (2024-07-06) showing increased abnormal signals and enhanced areas in the brain, with a larger and more pronounced enhancement than in the previous scan.

Figure 19: Chest CT (2024-07-05) showing an increase in diffuse, multiple punctate, millet-like, and small patchy high-density shadows in both lungs, with some areas enlarged compared to earlier imaging.

Figures 20-21: Cranial MRI (2024-08-13) showing a marked increase and enlargement in abnormal enhancement areas in the brain compared to the MRI on 2024-07-05.
